# Early acquisition of [^18^F]FDOPA PET/CT imaging in patients with recurrent or residual medullary thyroid cancer is safe—and slightly better!

**DOI:** 10.1186/s41824-022-00140-7

**Published:** 2022-08-25

**Authors:** Mette Louise Gram Kjærulff, André H. Dias, Peter Iversen, Lars Christian Gormsen, Karin Hjorthaug

**Affiliations:** Department of Nuclear Medicine and PET Centre, Aarhus University Hospital, Palle Juul-Jensens Boulevard 165, 8200 Aarhus N, Denmark

## Abstract

**Purpose:**

The aim of this study was to compare early (15 min) and late (60 min) [^18^F]FDOPA PET/CT acquisition times in the detection of recurrence/residual disease in medullary thyroid cancer (MTC) patients.

**Materials and Methods:**

Thirty-two dual-phase [^18^F]FDOPA PET scans were retrospectively reviewed. Scan indications were (1) suspected recurrence of MTC, (2) treatment monitoring, or (3) restaging. In four scans, no final verification could be obtained, and one scan was excluded due to non-consistency with the acquisition protocol. Images were analyzed visually and semiquantitatively (using SUV_max_). On both per-scan and per-lesion basis, early (median time 15 min) and late (median time 60 min) acquisition were compared by number and SUV_max_ of detected MTC lesions, and a washout rate between the two acquisitions was calculated. Sensitivity and specificity of early and late acquisition were also compared.

**Results:**

Out of the 27 eligible PET scans, twenty were classified as PET positive and 7 as PET negative. By subsequent histology and/or combination of imaging and clinical data during follow-up, the MTC diagnosis was verified, showing a scan-based sensitivity and specificity of 100% and 87.5%, respectively, for the early acquisition, and for the late acquisition both were 100%. However, there were no statistically significant difference in detection rate between the two acquisitions. Lesions on the early acquisition were significantly more intense compared to lesions on the late acquisition (median washout rate of − 33% (− 57 to + 50%)).

**Conclusion:**

Our study confirms that it is safe to omit the late [^18^F]FDOPA PET/CT acquisition in the detection of recurrent/residual MTC.

**Supplementary Information:**

The online version contains supplementary material available at 10.1186/s41824-022-00140-7.

## Introduction

Medullary thyroid carcinoma (MTC) is a rare neuroendocrine thyroid cancer subtype that accounts for up to 5% of all thyroid cancers. MTC is derived from the parafollicular calcitonin-secreting cells of the thyroid, and in 25–30% of the patients, MTC is part of inherited multiple endocrine neoplasia type 2 (MEN2) syndromes (Cabanillas et al. 2016; Maia et al. 2017; Giovanella et al. 2020; Taralli et al. 2020; Treglia et al. 2013; Soussan et al. 2012). Metastases are commonly seen in cervical lymph nodes, and lymphadenopathy is often the first objective finding. Total thyroidectomy is the only curative treatment followed by active surveillance, including ultrasonography and tumor markers (calcitonin and carcinoembryonic antigen) to detect possible recurrence of MTC (Cabanillas et al. 2016; Maia et al. 2017; Taralli et al. 2020; Treglia et al. 2013) which occurs in about 50% of the patients (Taralli et al. 2020; Treglia et al. 2013). Negative prognostic factors for MTC are large tumors, metastases, and age (Maia et al. 2017).

Fluorine-18-fluorodihydroxyphenylalanine ([^18^F]FDOPA) positron emission tomography (PET)/computed tomography (CT) is a sensitive imaging modality to detect recurrent or residual disease after total thyroidectomy in MTC patients with increased tumor markers (Giovanella et al. 2020; Taralli et al. 2020; Soussan et al. 2012). Several image acquisition protocols have been proposed with varying time from injection of [^18^F]FDOPA to scan start (acquisition time), from a few minutes, up to around 90 min (Taralli et al. 2020; Treglia et al. 2013; Golubić et al. 2017; Caobelli et al. 2018). Traditionally, the timing of image acquisition has been 60–90 min post-injection (p.i.). The European Association of Nuclear Medicine (EANM) practice guideline for PET/CT in MTC (Giovanella et al. 2020) advocates 30–60 min acquisition time. However, the guideline also states that an additional PET scan may be warranted 15 min p.i., since MTC lesions often have a rapid [^18^F]FDOPA washout rate (WR). Early or late [^18^F]FDOPA acquisition has been examined by a few previous studies in order to define the most optimal acquisition time, with evidence pointing toward higher detection rate of MTC lesions in the early images (Taralli et al. 2020; Treglia et al. 2013; Soussan et al. 2012; Golubić et al. 2017; Caobelli et al. 2018). However, these studies have been characterized by large variation in acquisition times at both the early and late images, and a standardized protocol with precise and uniform acquisition times is therefore needed.

At our department, an acquisition protocol for dual-phase [^18^F]FDOPA PET/CT in MTC patients with recurrent disease has existed for some time, involving standardized acquisition times set at 15 and 60 min p.i., respectively. It was therefore the aim of this retrospective study to determine the optimal acquisition time to detect recurrent or residual MTC lesions by [^18^F]FDOPA PET/CT.

## Methods

### Study population

This retrospective study includes dual-phase [^18^F]FDOPA PET/CT scans performed at the Department of Nuclear Medicine and PET-Centre, Aarhus University Hospital, Denmark, between April 2016 and June 2021. All patients diagnosed with MTC and referred for [^18^F]FDOPA PET/CT due to one of the following reasons were eligible for inclusion: suspected recurrent or residual disease after thyroidectomy (*n* = 17), treatment monitoring (*n* = 10), or restaging due to imaging, biochemically, and/or histologically documented recurrence (*n* = 5). In addition, both the early and late image acquisition had to be available. Three patients underwent more than one [^18^F]FDOPA PET/CT scan due to monitoring of ongoing treatment of unresectable advanced and symptomatic MTC with multikinase inhibitors. The individual scans in those patients were included as separate cases because the disease status changed during time due to the treatment. The institutional review board at Aarhus University Hospital granted access to patient files. Individual patients’ consent was waived due to the retrospective nature of the study.

### Reference standard and index test

#### Reference standard

Positive PET findings were ultimately validated as true positive (TP) or false positive (FP), and negative PET results as true negative (TN) of false negative (FN). For this validation, histology and/or a combination of imaging and clinical data during follow-up, including serum calcitonin trend, was used as reference standard.

#### [^18^F]FDOPA PET/CT procedure

During the study period, [^18^F]FDOPA PET/CT scans were performed using three different PET/CT systems; from April 2016 through October 24, 2018, a Siemens Biograph TruePoint TrueV scanner was used (*n* = 5), whereas subsequent PET/CT scans were performed on either a GE Healthcare Discovery MI scanner (*n* = 9) or a Siemens Biograph Vision 600 scanner (*n* = 18). For each patient visit, the early and late acquisition were performed in the same scanner. Our department, and thus all scanner systems, are EARL-accredited. Additional file 1: Table S1 shows reconstruction specifications of the three PET/CT scanner systems used in this study.

Patients fasted for at least 6 h prior to tracer injection. [^18^F]FDOPA was synthesized using a standardized method at the PET Centre at Aarhus University Hospital as described by Andersen et al. (2021). After an intravenous injection of 393.8 ± 45.2 MBq [^18^F]FDOPA co-administered with saline, PET scans were performed at both 15 and 60 min p.i. from vertex cranii to mid-femur. Early images were acquired after a median time of 15 (range 15–19) min p.i., and the late images with a median time of 60 (range 55–69) min after injection with [^18^F]FDOPA. A low-dose CT scan was performed for anatomical correlation and attenuation correction. No patients were premedicated with carbidopa.

#### [^18^F]FDOPA PET/CT image analysis

PET images were anonymized and subsequently reviewed in random order by an experienced nuclear medicine physician blinded to the subjects’ supplementary imaging and clinical data. The two phases (early and late) were evaluated visually and semiquantitatively using the maximum standardized uptake value (SUV_max_). Any focal accumulation of [^18^F]FDOPA outside the normal distribution or above the surrounding normal tissue was considered pathological. If the PET image analysis showed at least one lesion with pathological uptake, the respective imaging acquisition was defined as positive. In both early and late phase, the number of all pathological lesions at each anatomical site was recorded. If multiple pathologic lesions were found at the same anatomical site, only the highest SUV_max_ value among those lesions was recorded for the respective site. The image analysis also determined a WR (in %) for each anatomical site by the calculation 100 * (late SUV_max _– early SUV_max_)/early SUV_max_. The results on both acquisitions were compared on a per-patient basis and per-lesion site basis.

### Statistical analysis

Continuous variables are reported as mean ± standard deviation (SD), or median (with range). Categorical variables are expressed as a percentage. Normal distribution of SUV_max_ in early and late phase was assessed using Q–Q plots and the Shapiro–Wilk test. McNemar’s test was used to compare sensitivity and specificity of early and late image acquisitions. A comparison of SUV_max_ was made using Wilcoxon signed-rank test for paired samples. The Kruskal–Wallis test was used to assess whether SUV_max_ differed based on anatomical site of the lesions. Statistical significance was set at *p* < 0.05. Data processing was performed using Stata (StataCorp. 2021. *Stata Statistical Software: Release 17*. College Station, TX: StataCorp LLC).

## Results

### Study population

**Fig. 1:**
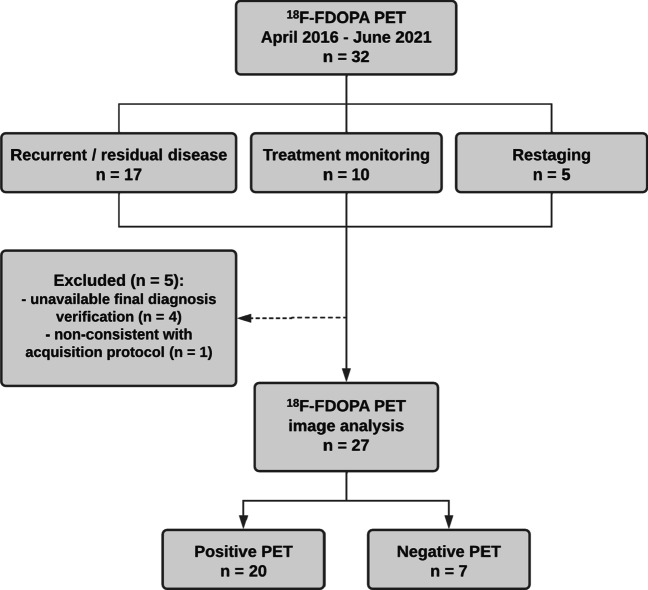
Flowchart showing the study population and [^18^F]FDOPA PET/CT results of included scans

Of the thirty-two [^18^F]FDOPA PET/CT scans initially selected for this retrospective study, four scans were excluded due to unavailable final verification of recurrent disease by the reference standard. In addition, a single scan was also excluded before analysis, as the early acquisition time was 27 min p.i. and thus almost twice as long as the acquisition protocol prescribed. Therefore, a total of 27 dual-phase scans (mean age at PET scan of 57 ± 13 (range 28–87) years) in 10 patients were included for further analysis. Image analysis concluded that 7 of the PET/CT scans were negative, and twenty PET/CT scans were defined as positive. In approximately half of the scans, the final diagnosis was confirmed by a combination of clinical data, biochemistry (serum calcitonin), and other imaging modalities such as magnetic resonance imaging (MRI), CT, and/or ultrasound during follow-up. Figure 1 shows a flowchart of the inclusion process, and clinical characteristics of the study population are summarized in Table 1. No correlation was found between serum calcitonin and SUV_max_ (tau-*b* = 0.110) or serum calcitonin and number of lesions (tau-*b* = − 0.228).

**Table 1:** Clinical characteristics of the study population

Characteristics	All scans (*n* = 27)	[^18^F]FDOPA uptake	
		Positive (*n* = 20)	Negative (*n* = 7)
Age, years (mean ± SD)	57 ± 13	59 ± 10	50 ± 17
Sex, male/female (%)	37/63	25/75	71/29
Serum calcitonin, pmol/L (median (range))	20.5 (0.29–262)^a^	62.0 (0.73–262)	5.9 (0.29–61.0)^b^
*Scan indication (in fractions):*			
Suspected recurrence	13/27	6/20	7/7
Treatment monitoring	10/27	10/20	0/7
Restaging	4/27	4/20	0/7
*Acquisition time, min (median (range))*			
Early	15 (15–19)	15 (15–19)	15 (15–18)
Late	60 (55–69)	60 (55–69)	60 (55–60)
[^18^F]FDOPA dose, MBq (mean ± SD)	393.8 ± 45.2	394.1 ± 52.6	393.0 ± 10.5
*Final diagnosis verification by reference standard (in fractions):*			
Histology + imaging*	8/27	8/20	0/7
Imaging* + clinical/biochemical data	15/27	8/20	7/7
Histology only	4/27	4/20	0/7

*SD* standard deviation

*MRI, CT and/or ultrasound

^a^one missing value (*n* = 26)

^b^one missing value (*n* = 6)

### Diagnostic accuracy of early and late acquisition

**Fig. 2:**
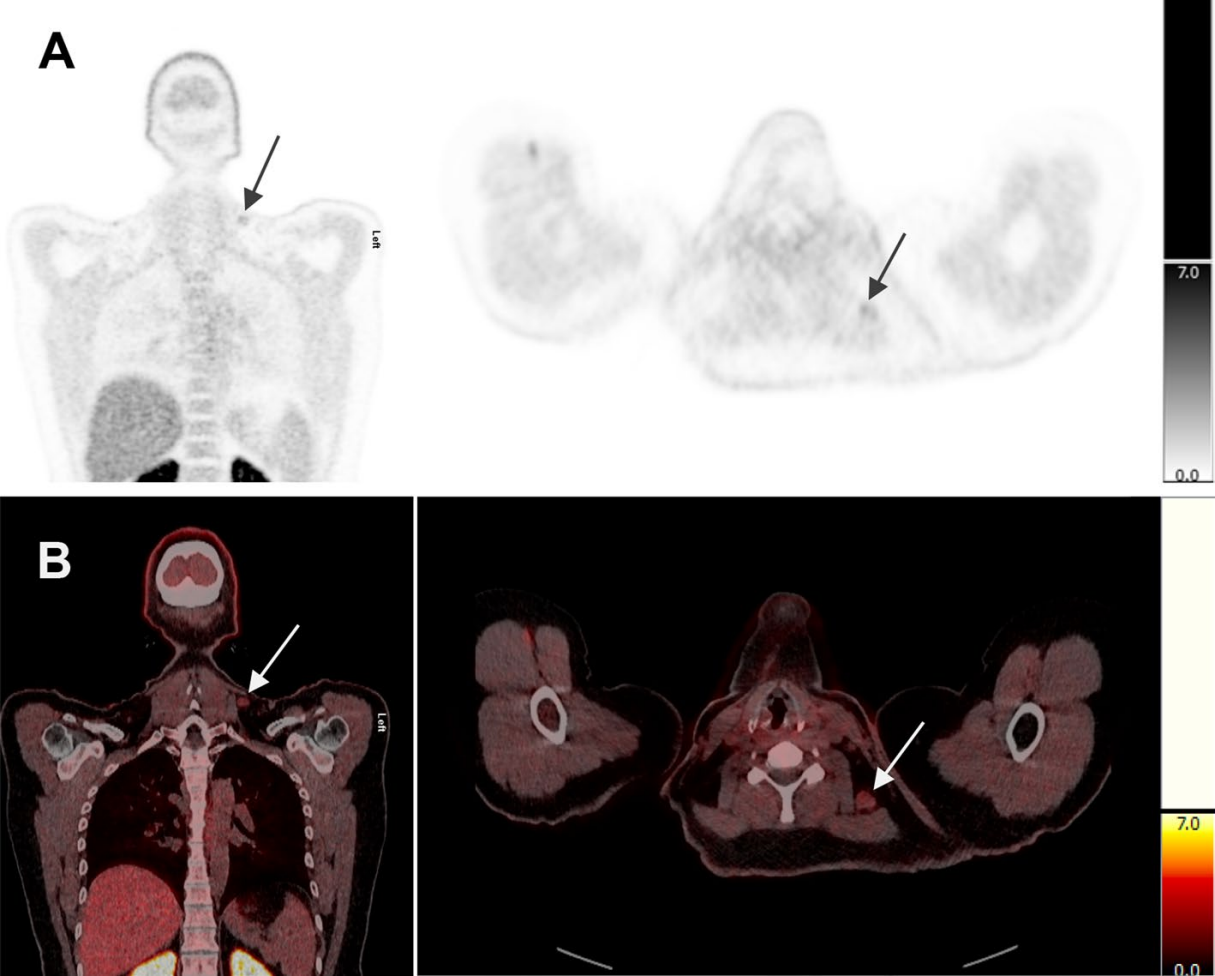
A 40-year-old woman (patient 9). Coronal and axial PET **A** and fused PET/CT **B** images showing slightly increased [^18^F]FDOPA uptake in a cervical lymph node (SUV_max_ = 2) on the early acquisition. Subsequent biopsy showed reactive changes and no malignancy

Final verification of the MTC diagnosis was obtained by the reference standard (cases by histology only, 8 cases by histology and imaging, and 15 cases by combination of imaging and clinical data at follow-up). Table 2 shows the verification distribution on a per-scan basis. The early acquisition presented a scan-based sensitivity of 100% and a specificity of 87.5%, whereas the late acquisition had a sensitivity and specificity of both 100%. Although the sensitivity of early and late acquisition was equal on *scan basis*, two scans showed lesions on the early acquisition, which were not detectable on the late acquisition (a total of 3 TP cervical lymph node lesions in 2 different patients). In a single patient case, increased FDOPA uptake was detected in a cervical lymph node on the early acquisition. The lesion was interpreted as pathological but a subsequent biopsy disproved this (false positive) (Fig. 2). There were no cases in which lesions were detected only on the late acquisition. However, the McNemar’s test showed no statistically significant difference between early and late acquisition on both per-scan and per-lesion site basis (*p* = 0.317 and 0.625, respectively).

**Table 2:** Summary of scan distribution

Early acquisition				
Reported findings	20	19 true positive	70.4%	Sensitivity = TP/TP + FN = 100%
		1 false positive	3.7%	
Remaining scans	7	0 false negative	0.0%	Specificity = TN/TN + FP = 87.5%
		7 true negative	25.9%	
Total	27		100%	

Late acquisition				
Reported findings	19	19 true positive	70.4%	Sensitivity = TP/TP + FN = 100%
		0 false positive	0.0%	
Remaining scans	8	0 false negative	0.0%	Specificity = TN/TN + FP = 100%
		8 true negative	29.6%	
Total	27		100%	

*TP* true positive, *FP* false positive, *TN* true negative, *FN* false negative

### SUV_max_ and washout rate

**Fig. 3:**
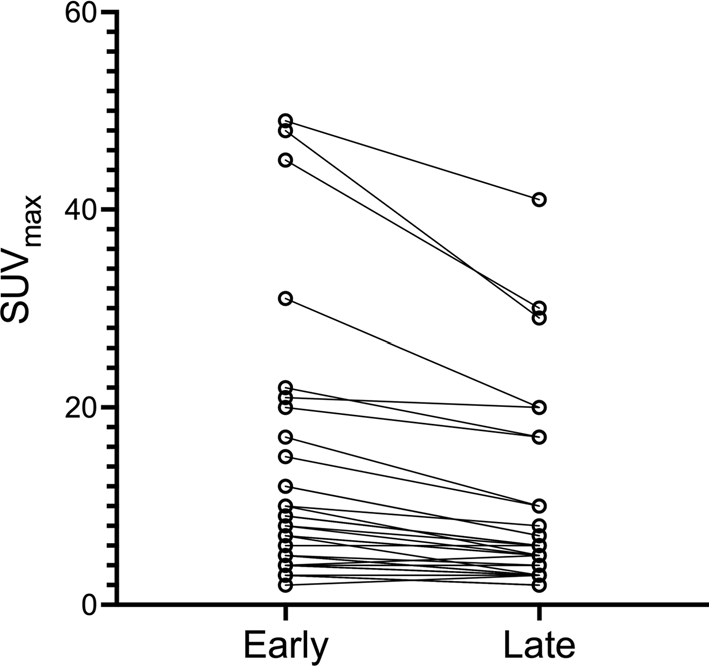
Difference in SUV_max_ (washout rate) between early and late acquisition (per-lesion site basis)

**Fig. 4:**
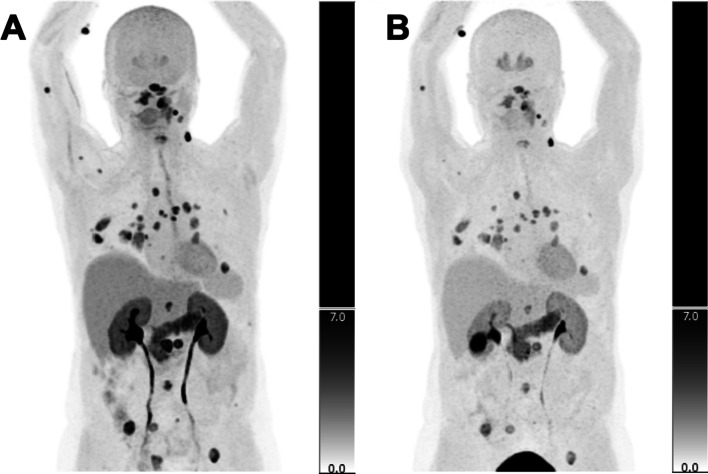
A 61-year-old woman (patient 1, case 4). **A** [^18^F]FDOPA uptake is more intense at the early acquisition 17 min p.i. compared to **B** the late acquisition at 58 min p.i

In only 2/38 TP lesion sites, a higher SUV_max_ was measured on the late acquisition compared to the early acquisition. This higher intensity of visualized lesions on the early acquisition was statistically significant (median SUV_max_ = 7.0 (range 2–48) on early vs. 5.0 (range 2–30) on the late phase, corresponding to a median WR of − 33% (− 57 to + 50%); *p* < 0.001). Figure 3 shows the difference in SUV_max_ between early and late acquisition on a per-lesion site basis, and Figs. 4 and 5 show scan examples of the more intense lesion visualization on early acquisition. The Kruskal–Wallis test showed no statistically significant difference in WR between different anatomical sites of TP lesions (*p* = 0.349). Additional file 2: Table S2 shows PET/CT results based on the various anatomical sites in the comparison of early and late acquisition.

**﻿Fig. 5:**
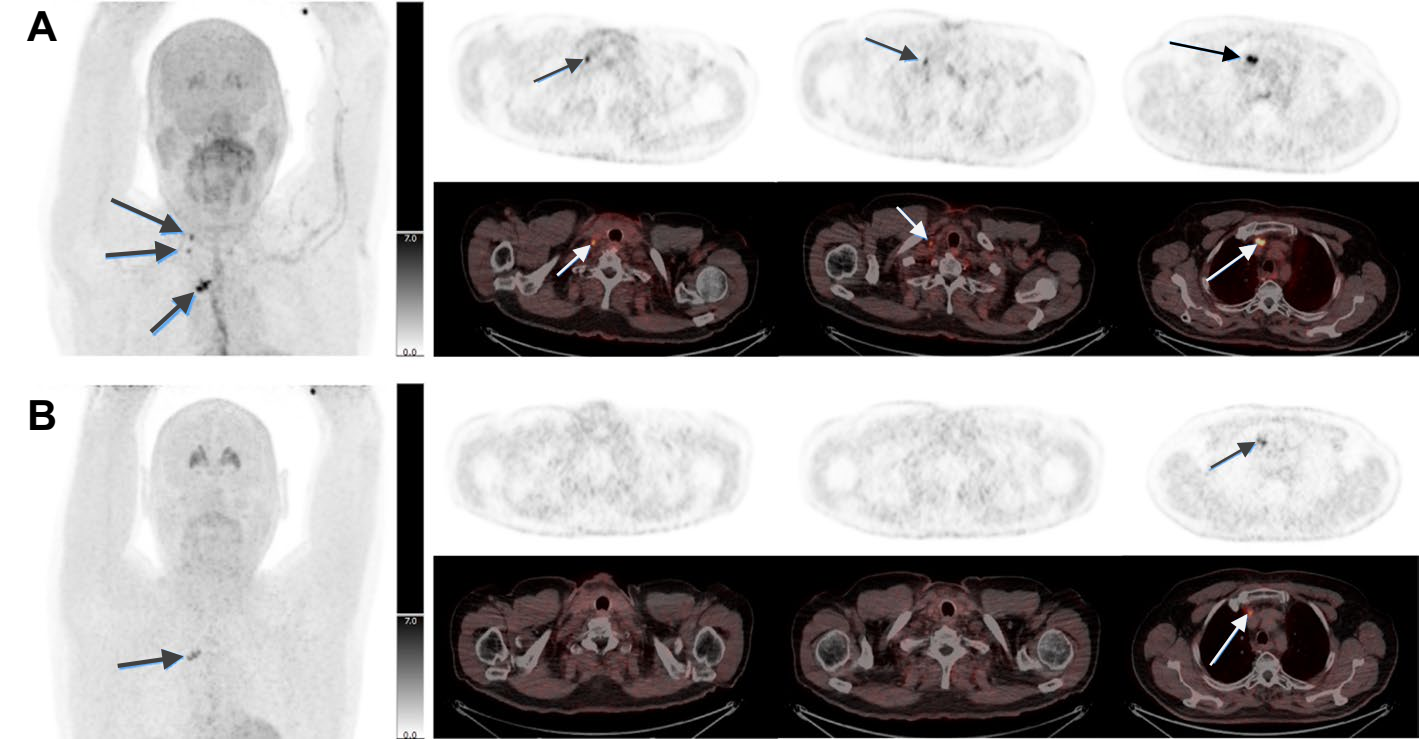
A 65-year-old man (patient 3). All lesions were verified by biopsies as true positive. **A** Imaging 15 min p.i. showed focal ^18^F-FDOPA uptake corresponding to two cervical lymph nodes (SUV_max_ = 6) and three thoracic lymph nodes (SUV_max_ = 10). **B** ^18^F-FDOPA uptake was no longer evident 59 min p.i. in the two cervical lymph nodes, and uptake in the thoracic lymph nodes was reduced (SUV_max_ = 5)

## Discussion

Our study demonstrates that MTC lesions on the early acquisition (15 min p.i.) are significantly more [^18^F]FDOPA avid than lesions on the late acquisition (60 min p.i.) with a median WR of − 33%. This confirms the results of previous studies (4–6), which also show a higher intensity of MTC lesions on early imaging. However, although lesions were more radiotracer avid on early acquisitions and 3 more TP lesions were found, we found no statistically significant difference in the detection rate of lesions between early and late acquisition. TP lesions that were only seen on the early images were all localized to cervical lymph nodes, suggesting that the rapid uptake and washout of [^18^F]FDOPA in MTC lesions, among other things, could be dependent on anatomical location. However, this hypothesis is not consistent with the study by Soussan et al. (2012), and in the current study, the range of WR in the lesions of the cervical lymph nodes is quite wide (− 41% to + 25%), and without a clear tendency specific for this anatomical site (Additional file 2: Table S2). This fits well with our statistical analysis, which shows no significant difference in WR between different anatomical sites of lesions. Thus, it can be concluded that location of MTC lesions has no significant impact on WR in our study. In a single patient, an FP lesion was detected only on the early acquisition (Fig. 2). The reference standard in this case was a biopsy, which described the lesion as a lymph node with reactive changes in the form of severe follicular hyperplasia and without malignancy-suspected cells. As mentioned in the EANM practice guideline for PET/CT in MTC (Giovanella et al. 2020), [^18^F]FDOPA uptake may in rare cases be due to inflammation which we assume is the reason for the activity uptake in our FP finding.

Several studies have investigated the value of [^18^F]FDOPA PET/CT in the detection of recurrent MTC, and as previously mentioned, these vary from a few minutes up to approx. 90 min in acquisition times (Taralli et al. 2020; Treglia et al. 2013; Soussan et al. 2012; Golubić et al. 2017; Caobelli et al. 2018). However, only a few studies have made a comparison of at least two acquisition times (Taralli et al. 2020; Treglia et al. 2013; Soussan et al. 2012) to obtain a time estimate for the most optimal (maximal) [^18^F]FDOPA uptake p.i., and only one of these is a dynamic study (Taralli et al. 2020). Among those studies, only the study by Treglia et al. (2013) reports similar acquisition times (15 and 60 min p.i.) as in our study, although they do not provide data on the exact acquisition times actually used or the variation of these between subjects. However, the WR observed by Treglia et al. (2013) is comparable to our result (mean WR − 28% ± 13% versus median WR − 33% (range − 57 to + 50%)). Soussan et al. (2012) observed a mean WR of − 40% but with widely varying acquisition times, especially the late acquisition which was obtained with a median time of 94 (range 70–150) min, making it difficult to compare with our standardized protocol. Standardized acquisition times are not only essential in the comparison of different studies; in treatment monitoring, where any disease progression or regression must be assessed, the scans should be performed with the same acquisition time protocol in order to be directly comparable. The dynamic study by Taralli et al. (2020) observed a median WR of − 41% (range − 88% to + 10%), which is not far from our WR value. However, their study is not directly comparable to ours both because the acquisition times are given in time intervals (2–5 min, 5–10 min, and 40–45 min, respectively) and because the early and late times do not match our exact times of 15 and 60 min, respectively.

In a clinical context, it is desirable to perform only one scan and the question is whether to do this early or late. In our study, none of the PET scans visualized lesions on the late acquisition that were not already detected on the early acquisition. Therefore, it can be assumed that no MTC lesions would have been missed if only the early acquisition had been performed. In previous studies (Taralli et al. 2020; Treglia et al. 2013) as well as in the present, no statistically significant difference was observed between the two acquisitions in the detection of MTC lesions on a patient/scan basis, indicating that the outcome for patients is generally the same whether only the early or only the late acquisition is performed. However, although the choice of either early or late acquisition did not have a clinical impact on patient outcome in our study, the scan-based median WR of -33% indicates (1) that it is safe to omit the late acquisition, and (2) that image acquisition, reading and reporting may in fact be simplified by doing so. In the scanning example in Fig. 5, the early acquisition visualizes two lesions in cervical lymph nodes that are not visible in the late image. In addition, activity uptake in three other lesions (thoracic lymph nodes) is seen to persist, albeit reduced, from early to late acquisition. These three lesions might as well have had a higher WR and have been washed out on the late image, as well as the two cervical lymph node lesions. Therefore, if no early imaging had been performed in such a case, the patient would have been visually declared free of MTC lesions. If only a late acquisition is made, a situation may therefore potentially arise where FN results are obtained.

Other studies have indicated that the early acquisition may be performed very shortly after radiotracer injection. Thus, Taralli et al. (2020) observed a rapid maximal [^18^F]FDOPA uptake after only a few minutes. Such short interval between injection and scan may simplify patient logistics considerably.

Our study is the first to apply whole-body acquisition in both the early and late phases, providing us the opportunity to analyze WR for several different anatomical sites in addition to the neck and thorax, which is a strength of the study. As for the study limitations, some may see it as a drawback that multiple scans from the same patient are included as separate cases, which could potentially lead to data distortion. Patients may have different biological behaviors in their metastases and thus different WR of [^18^F]FDOPA. A patient with several scans could therefore affect the overall result more, than a patient who has only had one scan. In our study, however, we do not consider this as a limitation, as the WR variation in general is wide. When looking at the same type of anatomical lesion site, this variation applies both interindividually and intraindividually when comparing scans from the same patient. We therefore do not believe that results from one patient with multiple scans are the basis for bias in our study. A second limitation of the study is that local tissue perfusion and blood volume may have an impact on the individual lesion washout rate. Clinically, however, it is difficult to deal with this limitation, as the perfusion can not only vary from patient to patient, but also between the individual patient’s visits in case of multiple scans. Thirdly, another limitation could be sample size. As mentioned, MTC is a rare type of cancer, and the previously mentioned studies (Taralli et al. 2020; Treglia et al. 2013; Soussan et al. 2012) include a sample size of between 15 and 21 scans. We therefore consider our sample size of 27 dual-phase scans to be relatively large in a rare type of cancer.

## Conclusion

In conclusion, [^18^F]FDOPA uptake was more intense in MTC lesions on the early (15 min p.i.) compared to the late (60 min p.i.) acquisition, and some lesions were only visualized on the early acquisition. There were no cases where lesions were only detected on the late acquisition. Even though no statistically significant difference in detection rate between early and late acquisition was observed, the findings of our study confirm that it may be safe to omit the later acquisition. A change in the 30–60 min acquisition time recommended in the EANM practice guideline for PET/CT in MTC (Giovanella et al. 2020) could therefore be beneficial.

## Supplementary Information

**Additional file 1**. **Table S1**: Reconstruction specifications of PET/CT systems.

**Additional file 2**. **Table S2**: Comparison between early and late acquisitions (*n* = 20). The table shows results based on the various anatomical lesion sites in the comparison of early and late acquisition.

## Data Availability

The datasets generated and analyzed during the current study are not publicly available due to ethical restrictions with patient data but are available from the corresponding author on reasonable request.

## Abbreviations

^18^F: Fluorine-18
CT: Computed tomography
EANM: European Association of Nuclear Medicine
FDOPA: Fluorodihydroxyphenylalanine
FN: False negative
FP: False positive
FWHM: Full width at half maximum
MEN2: Multiple endocrine neoplasia type 2
MRI: Magnetic resonance imaging
MTC: Medullary thyroid cancer
PET: Positron emission tomography
p.i.: Post-injection
SUV_max_: Maximum standardized uptake value
ToF: Time-of-flight
TN: True negative
TP: True positive
WR: Washout rate
