# Supplementary material for: Early acquisition of [18F]FDOPA PET/CT imaging in patients with recurrent or residual medullary thyroid cancer is safe—and slightly better!
Source: Eur J Hybrid Imaging. 2022 Aug 25;6:20. doi: 10.1186/s41824-022-00140-7 (PMC9402850; doi:10.1186/s41824-022-00140-7)
Supplement: Supplementary file 1 — Additional file 1. Table S1: Reconstruction specifications of PET/CT systems. [file 41824_2022_140_MOESM1_ESM.docx]

**Supplementary Table 1.** Reconstruction specifications of PET/CT systems

|  | Siemens Biograph Truepoint TrueV (n = 5) | GE Healthcare Discovery MI  (n = 9) | Siemens Biograph Vision 600 (n = 18) |
| --- | --- | --- | --- |
| Algorithm | TrueX  4 iterations, 21 subsets | VPFX SharpIR (with ToF)  3 iterations, 16 subsets | TrueX (with ToF)  4 iterations, 5 subsets |
| Filter | Gaussian filter of 3-mm FWHM | 2D Gaussian filter of 3-mm FWHM and ”light” axial filter | Gaussian filter of 2 mm |
| Matrix,  voxel size | 336 x 336,  2.0 x 2.0 x 2.0 mm^3^ | 384 x 384,  1.8 x 1.8 x 2.8 mm^3^ | 440 x 440,  1.65 x 1.65 x 1.65 mm^3^ |

*FWHM: full width at half maximum, ToF: time-of-flight*
