# Supplementary material for: Early acquisition of [18F]FDOPA PET/CT imaging in patients with recurrent or residual medullary thyroid cancer is safe—and slightly better!
Source: Eur J Hybrid Imaging. 2022 Aug 25;6:20. doi: 10.1186/s41824-022-00140-7 (PMC9402850; doi:10.1186/s41824-022-00140-7)
Supplement: Supplementary file 2 — Additional file 2. Table S2: Comparison between early and late acquisitions (n = 20). The table shows results based on the various anatomical lesion sites in the comparison of early and late acquisition. [file 41824_2022_140_MOESM2_ESM.docx]

**Supplementary Table 2.** Comparison between early and late acquisitions (n = 20).

| **P/S** | **Sex** | **Age** | **Acquisition times,**  **E/L (min)** | **Site** | **Early acquisition** | | **Late acquisition** | | **WR (%)** | **Serum-calcitonin (pmol/L)** | **Final diagnosis** |
| --- | --- | --- | --- | --- | --- | --- | --- | --- | --- | --- | --- |
|  |  |  |  |  | **Lesions (no.)** | **SUV_max_** | **Lesions (no.)** | **SUV_max_** |  |  |  |
| 1/1 | F | 60 | 15/60 | c.LN | Multiple | 45 | Multiple | 30 | -33 | 9 | TP |
|  |  |  |  | t.LN | Multiple | 8 | Multiple | 5 | -38 |  | TP |
|  |  |  |  | Bone | Multiple | 12 | Multiple | 7 | -42 |  | TP |
|  |  |  |  | Pleura | 1 | 3 | 1 | 3 | 0 |  | NA |
| 1/2 |  | 60 | 15/60 | c.LN | 5 | 48 | 5 | 29 | -40 | 8 | TP |
|  |  |  |  | t.LN | Multiple | 9 | Multiple | 6 | -33 |  | TP |
|  |  |  |  | Bone | Multiple | 12 | Multiple | 7 | -42 |  | TP |
|  |  |  |  | Pleura | 3 | 6 | 3 | 3 | -50 |  | NA |
| 1/3 |  | 61 | 17/58 | c.LN | 5 | 31 | 5 | 20 | -35 | 16 | TP |
|  |  |  |  | t.LN | Multiple | 9 | Multiple | 6 | -33 |  | TP |
|  |  |  |  | Lung | 5 | 7 | 5 | 3 | -57 |  | TP |
|  |  |  |  | Bone | Multiple | 15 | Multiple | 10 | -33 |  | TP |
|  |  |  |  | Pleura | 1 | 3 | 1 | 3 | 0 |  | NA |
| 1/4 |  | 61 | 17/58 | t.LN | Multiple | 4 | Multiple | 3 | -25 | 5.6 | TP |
|  |  |  |  | Bone | Multiple | 5 | Multiple | 3 | -40 |  | TP |
| 1/5 |  | 61 | 16/60 | t.LN | Multiple | 5 | Multiple | 3 | -40 | 6.6 | TP |
|  |  |  |  | Bone | Multiple | 7 | Multiple | 5 | -29 |  | TP |
| 1/6 |  | 62 | 15/60 | t.LN | Multiple | 5 | Multiple | 4 | -20 | 8.5 | TP |
|  |  |  |  | Bone | Multiple | 9 | Multiple | 6 | -33 |  | TP |
| 1/7 |  | 62 | 17/57 | t.LN | Multiple | 3 | Multiple | 2 | -33 | 7.9 | TP |
|  |  |  |  | Bone | Multiple | 3 | Multiple | 2 | -33 |  | TP |
| 2/8 | F | 74 | 16/59 | c.LN | 1 | 4 | 1 | 4 | 0 | 58 | TP |
|  |  |  |  | Lung | 1 | 4 | 1 | 2 | -50 |  | FP |
| 3/9 | M | 65 | 15/59 | c.LN | 2 | 6 | 0 | - | - | 242 | TP |
|  |  |  |  | t.LN | 3 | 10 | 3 | 5 | -50 |  | TP |
| 4/10 | F | 56 | 15/60 | Liver | 3 | 10 | 3 | 8 | -20 | 189 | TP |
|  |  |  |  | Bone | 1 | 3 | 1 | 3 | 0 |  | TP |
| 5/11 | F | 87 | 15/67 | c.LN | 2 | 5 | 1 | 3 | -40 | 259 | TP |
|  |  |  |  | t.LN | 1 | 4 | 1 | 3 | -25 |  | TP |
|  |  |  |  | Thyr.sin | 1 | 6 | 1 | 6 | 0 |  | TP |
| 6/12 | M | 71 | 15/63 | Bone | 5 | 4 | 5 | 4 | 0 | 262 | TP |
| 7/13 | M | 51 | 19/56 | c.LN | 2 | 8 | 2 | 6 | -25 | 66 | TP |
| 7/14 |  | 52 | 16/69 | c.LN | 2 | 9 | 2 | 6 | -33 | 98 | TP |
| 8/15 | M | 47 | 15/60 | c.LN | 2 | 4 | 2 | 5 | 25 | 25 | TP |
|  |  |  |  | t.LN | 1 | 3 | 0 | - | - |  | FP |
| 9/16 | F | 40 | 15/60 | c.LN | 1 | 2 | 0 | - | - | 0.73 | FP |
| 10/17 | F | 54 | 15/55 | c.LN | 3 | 17 | 3 | 10 | -41 | 163 | TP |
|  |  |  |  | Lung | 1 | 3 | 1 | 2 | -33 |  | TP |
| 10/18 |  | 54 | 15/60 | t.LN | 3 | 20 | 3 | 17 | -15 | 168 | TP |
|  |  |  |  | Lung | 1 | 3 | 1 | 3 | 0 |  | TP |
| 10/19 |  | 55 | 16/58 | t.LN | 3 | 21 | 3 | 20 | -5 | 178 | TP |
|  |  |  |  | Lung | 1 | 2 | 1 | 3 | 50 |  | TP |
| 10/20 |  | 55 | 15/58 | t.LN | 3 | 22 | 3 | 17 | -23 | 123 | TP |
|  |  |  |  | Lung | 1 | 4 | 1 | 3 | -25 |  | TP |

P/S: patient/scan, WR: washout rate, M: male, F: female, E/L: early/late, t.LN: thoracic lymph node, c.LN: cervical lymph node, TP: true positive, FP: false positive, NA: Not available.
